# Household Hints and Recipes

**Published:** 1882-01

**Authors:** 


### Household Hints & Recipes

TO CLEAN OLD MARBLE OR ALABASTER.—Immerse the objects for two to three days in water to soften the dirt, lime, etc., then take them out and clean them with a brush or scraper. When cleaned in this way as much as possible, lay them in a mixture of one part of concentrated muriatic acid and three parts of water until they appear perfectly clean. Sometimes it may be necessary to increase the "biting" property of the sour water with nitric acid. Finally, soak the objects in water till they are perfectly free from acid. The appearance may often be improved by rubbing with a little almond oil.

IMITATION OF GROUND GLASS.—The following, if neatly done, renders the glass obscure yet diaphanous: Rub up, as for oil colors, a sufficient quantity of sugar of lead with a little boiled linseed oil, and distribute this uniformly over the pane, from the end of a hog-hair tool, by a dabbing, jerking motion, until the appearance of ground glass is obtained. It may be ornamented, when perfectly hard, by delineating the pattern with a strong solution of caustic potash, giving such time to act as experience dictates, and then expeditiously wiping out the portion it is necessaɹy to remove.

SPRUCE BEER.—One mode of preparing this is as follows: Take water, 16 gallons, boil half, put the water thus boiled to the reserved cold half, which should be previously put into a barrel or other vessel; then add 16 lbs. of treacle, or molasses, with a few spoonfuls of essence of spruce, stirring the whole together; add half pint of yeast, and keep it in a temperate situation with the bung hole open for two days, or till fermentation subsides; then close it up or bottle it off, and it will be fit to drink in a few days. This is a cheap and wholesome drink, and is much recommended for internal injuries.

TO PRESERVE WOOD POSTS.--The decay of wood embedded in the earth is difficult to guard against; but, according to the *Farmers' Gazette*, a simple precaution, costing neither money nor labor, will increase the durability of posts put in the ground by fifty per cent. This is simply by taking care that the wood is inverted, *i. e.*, placed in the opposite direction to that in which it grew. Experiments have proved that oak posts put in the ground in the same position as that in which they grew, top upwards, were rotten in twelve years, while their neighbors, cut from the same tree and placed top downwards in the soil, showed no signs of decay for several years afterwards. The theory is that the capillary tubes in the tree are so adjusted as to oppose the rising moisture when the wood is inverted.

REMOVAL OF GREASE SPOTS.—Fatty oils have a greater surface-tension than oil of turpentine, benzole, or ether. Hence, if a grease spot on a piece of cloth be moistened on the reverse side with one of these solvents, the tension on the greasy side is larger, and therefore the mixture of benzole and fat or grease will tend to move towards the main grease spot. If we were to moisten the center of this spot with benzole, we should not remove it, but drive the grease upon the clean portion of the cloth. It is, therefore, necessary to distribute the benzole first over a circle surrouuding the grease spot, to approach the latter gradually, at the same time having blotting paper in contact with the spot to absorb the fat immediately.

Another method, namely to apply a hot iron on one side, while blotting paper is applied to the other, depends upon the fact that the surface-tension of a substance diminishes with a rise of temperature. If, therefore, the temperature at different portions or sides of the cloth is different, the fat acquires a tendency to move from the hotter parts towards the cooler.—*Maxwell, in the Pharmacist.*

THE PROFILOGRAPH.—A new device has been brought out called the "profilograph," intended, as the name indicates, to trace automatically the profile of a road or district. It consists of a two-wheeled carriage, from the body of which is suspended a pendulum, free to swing in a line with the direction in which the carriage moves. It retains always, of course, a vertical position, whatever the inclination of the carriage traveling over uneven ground may happen to be. The upper end of the pendulum above the point of support carries a pencil which touches a ribbon of paper moved by clockwork, or actuated by the movement of the wheels of the carriage. As long as the latter is moving, a profile of the country passed over is traced on the paper. At the same time one of the wheels, by a simple pedometric device, records the distance traversed and makes a scale for comparison with the profile tracing, to show the relation of the two measures of height and distance passed over by the machine.

